# Clinical Remarks on Cases of Acute Necrosis

**Published:** 1877-01

**Authors:** 

**Affiliations:** St. Bartholomew's Hospital.


### ARTICLE VI.

### *Clinical Remarks on Cases of Acute Necrosis.*

BY MR. SAVORY, OF ST. BARTHOLOMEW'S HOSPITAL.

Among the subjects treated of by modern writers on surgery, necrosis is one that has received less attention than it deserves. Necrosis is of two forms—acute and chronic. By chronic necrosis is understood such an affection of bone as is constantly seen and operated upon with success in the young adult. Its ordinary course is, that from some cause or other a portion of the bone perishes. As a result of this an abscess forms, which is either opened by the surgeon or breaks spontaneously. On a probe being passed through the opening, the dead bone is at once detected. A portion of bone having perished, new bone is formed; the dead portion becomes loose, and remains inclosed in a cavity formed by the new bone. The duration of each case depends on the working out of these results.

Acute necrosis presents features quite different from these. It is a disease the true nature of which has only been recognized in recent times. As here applied, the term "necrosis" is an unfortunate one, for it takes into account only the final result, and not the nature of the affection. It would be better to lay it aside, and substitute "ostitis."

Necrosis is commonest in the shafts of the long bones, because there the bone is densest, and consequently there the vessels are most readily strangled when inflammation takes place. Caries is commonest in the cancellous tissue, because there inflammation can run a longer course without the circulation being so far impeded as to cause death in mass. Dr. Wilks, of Guy s Hospital, years ago published a paper in the *Guy's Hospital Reports*, on "Deep seated Abscess of the Limbs;" and the cases there mentioned, looked at with the more thorough knowledge of to-day, are evidently cases of acute necrosis, accompanied, as these always are, by deep-seated abscesses.

To say that periostitis is the cause of acute necrosis is only to tell a part of the truth; so, too, it is an error to affirm that osteo myelitis alone is its cause. Inflammation of bone, accompanied by both periostitis and osteo-myelitis, is its cause.

With regard to the causes of ostitis: The extension of in-flammation from surrounding parts is rare, but may occasionally take place. Scrofula, whatever that obscure constitutional condition may be, must be regarded as predisposing to it. Syphilis is said to be a predisposing cause; but this is very doubtful. The early age at which it occurs negatives the existence of acquired syphilis; and I have not seen the hereditary affection in connection with it. Violence is a frequent cause; but the most frequent is exposure to cold and wet, as is exemplified in one of the cases upon which these remarks are founded. It is also very liable to follow where the vital powers of a patient have been much reduced by fever; thus, it sometimes follows scarlet fever, as it did in the second case brought foward.

A typical case has the following course: A delicate child (for it is a disease almost confned to children) is seized with severe pain in the leg or thigh. On examination, we find an undefined swelling, the bone and surrounding parts being fused together. There are the symptoms of local inflammation and severe surgical fever. Rigors are present, there is considerable elevation of temperature, and increased frequency of pulse. In a few days obscure fluctuation becomes appreciable in the swelling, and if now an opening be made a probe can be passed down to dead bone. During these few days the constitutional symptoms increase in severity. The after-progress of the case is marked by profuse suppuration; hectic takes the place of the surgical fever; and the child may die of exhaustion or pyæmia, or may recover with the limb so far damaged that new bone has replaced the old.

The first case is that of a boy, Roberi L., aged ten years. Five months ago, while playing, he strained himself. The strain was followed by aching pain and limping. In the first week in October he got wet ; a week afterwards there were swelling and pain in the thigh. The whole limb was erected, and shorter than the other by an inch and a half. The swelling went on to the formation of abscess, which was opened on the 11th. The temperature at one time rose as high as 106°.

As a rule, acute necrosis in the child affects the entire shaft of a bone, the epiphyses escaping, because in childhood they are distinct from the shaft. If they do not escape, the case is desperate ; the joint is sure to become involved. In the above case it is doubtful whether the head of the bone became affected with necrosis, or whether the dislocation resulted from disease of the hip which had previously existed. The history of the case rather bears out the latter supposition.

The second case is that of a girl, Louisa F., also aged ten years. After an attack of scarlet fever, followed by abscess under the side of the chin, the right knee is described as becoming affected with acute synovitis. This subsided in about three weeks, but some swelling remained on the inner side of the thigh, where also there was doubtful fluctuation. Poultices were ordered, an abscess formed and was opened, and dead bone was detected with the probe. Subsequently erysipelas supervened and spread from the wound upwards. This is usually the direction which the disease takes ; but in this case, after spreading upwards for some time, it changed its course and went downwards.

When necrosis has taken place, the sources of new bone in the adult are the periosteum, the living remains of the old bone, and the surrounding soft parts. But for the regeneration of bone in the child one of these is lost, for the periosteum perishes from the fierceness of the inflammation. The specimen shown should be looked upon as one of the most valuable in the museum, for it shows this point very well. It is the clavicle of a child, which, with its periosteum, has entirely perished from this disease.

The epiphyses are capable of playing a very important part in the production of new bone. In two cases where the entire shaft had perished, I have seen its reproduction brought about by the growth of new bone upward and down ward from them till it has met in the centre.

A few words may be said respecting the occurrence of pyæmia in these cases. As a cause of this, acute necrosis is second only in frequency to wounds; and I fancy pericarditis accompanies cases of pyæmia resulting from acute necrosis oftener than from other causes.

With regard to treatment, it is best to cut down at once, and deeply, to the bone. By doing this the infiltration of surrounding parts and the destruction of the periosteum are prevented. In practice, amputation is rarely performed to avoid pyæmia. So long as that is only a possible event, it would not be justifiable, but when it threatens it would be right. Generally the indications for amputation depend on the part of the bone affected. So long as the shaft alone is diseased, so long we may hope to do without it; but when the epiphyses become involved, it only remains to choose the most fitting time for its performance.—*Med. Times and Gaz.*

